# The Stroop Task Influences Product Evaluations

**DOI:** 10.3389/fpsyg.2021.688048

**Published:** 2021-07-15

**Authors:** Tom G. E. Damen

**Affiliations:** Department of Psychology, Utrecht University, Utrecht, Netherlands

**Keywords:** product preferences, cognitive conflict, Stroop task, goal relevance, conditioning

## Abstract

Cognitive conflict is considered to represent a psychologically negative signal. Indeed, a recent publication showed that cognitive conflict emerging from the Stroop task influences evaluations for neutral shapes that had become associated with conflict and non-conflict, respectively. Building on these findings, the present research investigates the degree to which Stroop conflict influences evaluations of actual products. In an experimental study, participants performed a Stroop task in which they responded to conflict trials (e.g., the word red presented in a blue font) as well as non-conflict trials (e.g., the word red presented in a red font). Participants were also presented with two pictures featuring bottled water brands: One brand was consistently presented after non-conflict trials; the other brand was consistently presented after conflict trials. When participants evaluated the products, the results showed they rated the product associated with Stroop conflict less favorably than the product associated with non-conflict; however, this effect only emerged when participants were thirsty. When participants were not thirsty, no differences emerged. The present findings add to the literature on cognitive conflict and negativity, suggesting that Stroop conflict can influence product evaluations when those products are goal relevant.

## Introduction

How do product preferences emerge? Each day, our consumer society provides an avalanche of product choices. But how do we make a choice between different jeans, and how do we know if we want a Pepsi and a Coke, or whether we will like red velvet or a blueberry cheesecake? Typically, we will use affective information from previous experiences when determining how we feel about something, or what to buy (Schwarz, 1997; Luce et al., 1999; Phillips and Baumgartner, 2002). For example, whether or not we were happy with a specific product in the past strongly determines which products we prefer in the future (Fazio, 1990). However, affect may also shape our evaluations in less obvious ways. For example, a decade’s worth of research in the domain of experimental psychology showed that negative affect emerges when mental processes are conflicting (see reviews by Dreisbach and Fischer, 2015; Inzlicht et al., 2015; van Steenbergen, 2015; Dignath et al., 2020). This was illustrated in a recent publication by Damen et al. (2018) who showed that the negativity evoked by cognitive conflict emerging on a Stroop task “spilled over” to influence evaluations of polygon shapes. But does Stroop conflict negativity also influence our preferences for actual products? And how important is it for those products to be relevant to our needs? The goal of the present research was to answer those questions.

### The Stroop Effect

The Stroop effect (Stroop, 1935) refers to our tendency to experience difficulty when naming a word’s physical color when that word spells the name of a different color. It is relatively difficult to declare that a word is presented in a blue hue, when that word in fact reads “red.” This task poses a challenge because reading is a relatively automatized process that in this instance interferes with color perception (MacLeod, 1991; MacLeod and MacDonald, 2000). Trials on which Stroop conflict emerges typically lead individuals to make more errors and have longer response times compared to conditions of non-conflict (Cohen et al., 1990; MacLeod, 2005). This Stroop effect represents one of the most robust and reliable effects in experimental psychology (MacLeod, 1992; Ebersole et al., 2016).

### Conflict Negativity

It is long theorized that cognitive conflicts, such as the conflicts emerging on the Stroop task, are experienced as negative signals (Botvinick, 2007; Inzlicht et al., 2015). In fact, conflict negativity may be instrumental in recruiting the mental resources required to cognitively resolve the conflict. Strong support for the notion of conflict as a negative signal comes from research that showed that individuals who perform the Stroop task also exhibit neural activity in brain regions that are associated with negativity (MacDonald et al., 2000; Kerns et al., 2004; Vermeylen et al., 2020). Other research that more directly investigated the conflict negativity hypothesis revealed that individuals who were exposed to Stroop conflict more quickly categorized subsequent negative words as negative (Dreisbach and Fischer, 2012), or were more likely to assign negative labels to subsequent stimuli (Fritz and Dreisbach, 2013; see also Goller et al., 2017).

A recent paper by Damen et al. (2018) reported on 11 studies in which participants were presented with conflict and non-conflict Stroop color words followed by neutral polygon shapes. In 10 out of the 11 studies, participants explicitly rated the polygons following conflict Stroop words less favorably compared to polygons following non-conflict Stroop words. This effect emerged robustly, independent from a number of methodological variations. A meta-analysis over this collection of studies in fact indicated a reliable effect with a small to medium effect size, thereby providing further support for the conflict negativity hypothesis (Damen et al., 2018).

The process through which conflict influences the evaluations of associated items may reflect a form of evaluative conditioning (EC; e.g., De Houwer et al., 2001; Hofmann et al., 2010). Research on EC has shown that when one stimulus is paired with another negative stimulus, evaluations of that former stimulus become more negative. For example, when the picture of a human face is paired with the picture of a grotesque wound, or with a negative word such as “death,” that human face is subsequently liked less than before (and similarly, positive associations make the face to be liked more; Levey and Martin, 1975; Baeyens and De Houwer, 1995). Conflict negativity provides a novel take on EC in the sense that the associated valence does not emerge from inherently negative pictures or word meaning, but because mental processes yield conflicting information. However, while EC has been well established as a process through which product evaluations emerge and change (e.g., Gibson, 2008; Stahl et al., 2009; Sweldens et al., 2010), whether similar results emerge from cognitive conflict is unclear. Addressing this gap would provide valuable knowledge about the applicability of Stroop conflict as a tool for attitude change. It would also provide additional insights as to whether results change when investigating meaningful and relevant stimuli, such as consumer products, as opposed to neutral or ambiguous stimuli, such as polygon shapes (Damen et al., 2018).

Which products we attend to, and which products we like or dislike, is strongly determined by the goal relevance of those products. A compelling body of research reveals that goals exert strong influences on perceptual, cognitive, and evaluative processes (Aarts and Dijksterhuis, 2000; Shah et al., 2002; Ferguson and Bargh, 2004). For example, goal-relevant information is more easily recognized (Gollwitzer, 1999), and goal-relevant knowledge is more easily retrieved (Aarts et al., 2001; Moskowitz, 2002), whereas concepts that might obstruct goal pursuit are inhibited (Shah et al., 2002). Furthermore, goal-relevant stimuli are automatically evaluated more positively than irrelevant stimuli (Ferguson and Bargh, 2004; see also Spruyt et al., 2018). Finally, it was shown that the magnitude of EC and subliminal priming effects is modulated by goal and product relevance (respectively, Verwijmeren et al., 2012; and Karremans et al., 2006). As such, how we perceive and evaluate products is often very dependent on whether or not we have a biological or social goal that those products allow us to achieve. Given the literature on goal relevance, it is not only possible but even likely that cognitive conflict will especially influence evaluations for products when those products are goal relevant.

### The Present Research

The aim of the present study was to replicate previous findings showing that the Stroop task can influence evaluations of associated stimuli (Damen et al., 2018). Furthermore, it also aimed to expand the research in this domain by investigating the generalizability and applicability of Stroop (non-)conflict as a tool to influence evaluations of consumer products. A final aim was to explore goal relevance as an important moderator of the conflict negativity effect when in a consumer context.

An experimental study was conducted in which participants performed the Stroop task. Participants responded to both non-conflict trials (e.g., the word red presented in a red font) and conflict trials (e.g., the word red presented in a blue font). Participants were also presented with pictures featuring different brands of bottled water: One brand was consistently presented after non-conflict trials; the other brand was consistently presented after conflict trials.

There were three main hypotheses. First, it was expected that the product associated with the compatible non-conflict trials would be evaluated more favorably compared to the product that was associated with the incompatible conflict trials. Second, it was expected that both products would be evaluated more favorably when participants were thirsty compared to when they were not thirsty. Third, an interaction effect between Stroop compatibility and product relevance was explored. If such an interaction emerged, it was expected that a compatibility effect on product evaluations would be stronger when participants were thirsty.

## Materials and Methods

### Participants

Sixty-six Dutch nationals (40 males; *M*_age_ = 33.77) participated in exchange for a small fee. Participants were recruited through Prolific.ac, an integrated participant recruitment and compensation system that is both diverse and reliable. Studies were conducted using the online environment of Inquisit 4.0.2. A power analysis (G*Power; Faul et al., 2009) indicated that this sample size was proper given an analysis involving two within-subjects levels, a 5% alpha-level, 80% statistical power, correlations among repeated measures >0.65, and an effect size of *f* = 0.44 [i.e., the correlation among repeated measures and the effect size parameters were based on Damen et al. (2018)]. This sample size also provided enough power to reliably detect within-between interaction effects of *f* = 0.14, meaning that the analysis will be sensitive enough to detect relatively small effects. The research was approved by the Utrecht University’s Faculty Ethics Review Board.

### Task

Participants performed a mouse click Stroop task (Linnman et al., 2006). They were told that at the start of each trial, they would see words appearing on their monitor in one of four colors: “green,” “yellow,” “purple,” and “red.” Participants were instructed to indicate as quickly and accurately as possible the color in which the word was presented by clicking the appropriate response button with their computer mouse. Participants performed four practice trials. Two practice trials featured Stroop compatibility (e.g., the word red presented in a red font) – no conflict should emerge on these trials. Two other practice trials featured Stroop incompatibility (e.g., the word red presented in a yellow font) – these represent conflict trials. There was no response deadline. After completing the practice trials, participants proceeded to the main task.

Trials on the main task were identical to the practice trials except for the fact that after each response, participants were presented with a picture of a specific product. Participants were instructed to look at the product pictures as there would be questions about them at the end of the study. The picture either featured a bottle of the mineral water brand “Dasani” or a bottle of the brand “Deja Blue.” Neither brand has ever been distributed in Netherlands.^1^ In a follow-up query to which 56 out of 66 participants responded, no single participant indicated familiarity with either of the brands. Randomly determined, one of the products was consistently presented for 1,000 ms after compatible Stroop trials, and the other product was consistently presented after the incompatible Stroop trials. If a wrong response was given on the Stroop task, no picture was presented and the trial was restarted (2% of the trials).^2^ After the presentation of the product picture, there was an intertrial pause of 1,200 ms. The main task featured 20 compatible and 20 incompatible Stroop trials which were selected randomly without replacement.

**Figure 1:**
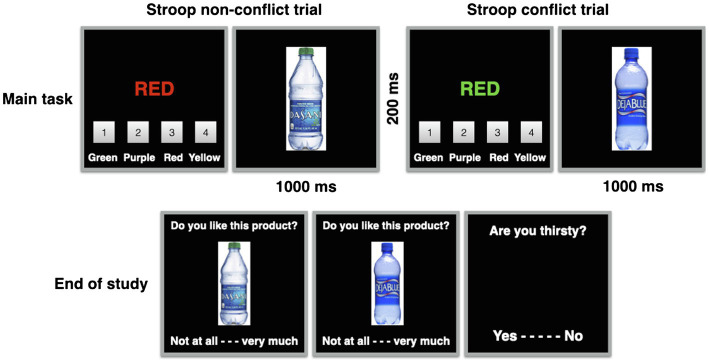
Experimental overview. The upper half depicts a compatible non-conflict trial and an incompatible conflict trial, respectively. The lower half depicts the evaluations at the end of the study.

At the end of the study, participants were again shown the product pictures and for each product were asked to evaluate the products on a scale of 1–9 (1 = *Extremely disliked*; 9 = *Extremely liked*). Participants were then asked to indicate whether or not they were thirsty (thirsty vs. not thirsty).^3^ Thirty-five out of 66 participants indicated they were thirsty. Finally, participants were thanked and debriefed. The study procedure is visualized in Figure 1.

The study was reviewed and approved by the Faculty Ethics Assessment Committee – Social Sciences, Utrecht University. The participants provided their written informed consent to participate in this study.

## Results

### Stroop Effect

A two-level (Stroop compatibility: compatible vs. incompatible) repeated measures ANOVA on the number of incorrect responses showed that participants made more errors on incompatible trials than on compatible trials {*M*_Compatible_ = 0.046, *SD* = 0.274 vs. *M*_Incompatible_ = 0.379, *SD* = 0.674; *F*(1, 65) = 13.750, *p* < 0.001, $\eta_{p}^{2}$ = 0.175 [CI_95%_ 0.038–0.331]}. For the analysis on reaction times (RTs), responses shorter than 400 ms and longer than 4,000 ms were set at 400 ms and 4,000 ms, respectively. The analysis on RTs showed that participants were slower to respond to the incompatible targets compared to the compatible targets {*M*_Compatible_ = 1,345, *SD* = 324 vs. *M*_Incompatible_ = 1,568, *SD* = 406; *F*(1, 65) = 76.645, *p* < 0.001, $\eta_{p}^{2}$ = 0.541 [CI_95%_ 0.369–0.652]}. This replicates the classic Stroop (1935) effect.

### Product Evaluations

**Figure 2:**
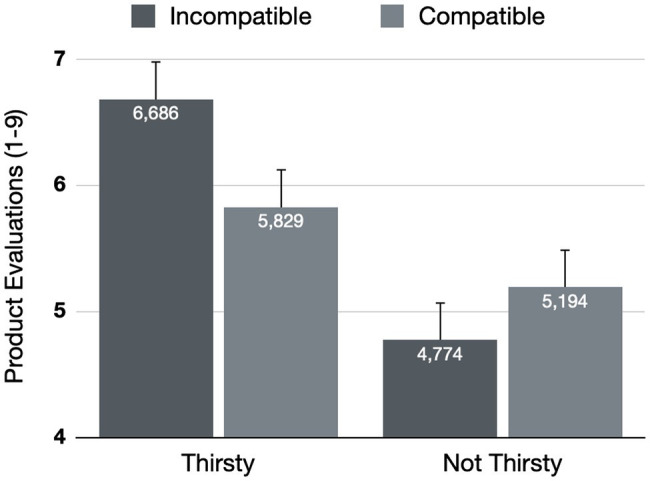
Product evaluations as a function of Stroop compatibility associations and thirstiness. Error bars denote SEs.

Results on product evaluations are visualized in Figure 2. A 2 (Stroop compatibility: compatible vs. incompatible) × 2 (Thirsty: yes vs. no) repeated measures ANOVA on liking scores showed that participants did not rate the product paired with the compatible Stroop trials differently from the product paired with the incompatible Stroop trials {*M*_Compatible_ = 5.788, *SD* = 2.130 vs. *M*_Incompatible_ = 5.530, *SD* = 1.994; *F*(1, 64) = 1.085, *p* = 0.301, $\eta_{p}^{2}$ = 0.017 [CI_95%_ 0.000–0.120]}. Results did however show a main effect of thirst, as the products in general were evaluated more favorably when they were thirsty {*M*_Thirsty_ = 6.257, *SD* = 1.574 vs. *M*_Not thirsty_ = 5.063, *SD* = 1.958; *F*(1, 64) = 8.662, *p* = 0.005, $\eta_{p}^{2}$ = 0.119 [CI_95%_ 0.012–0.271]}. Most importantly, however, is that these effects were qualified by a significant interaction between the Stroop compatibility and Thirst conditions {*F*(1, 64) = 9.225, *p* = 0.003, $\eta_{p}^{2}$ = 0.126 [CI_95%_ 0.015–0.279]}.

Decomposing the interaction, simple contrasts showed that when participants were thirsty, they evaluated the product associated with the compatible trials more favorably than the product associated with the incompatible trials {*M*_Compatible_ = 6.686, *SD* = 1.827 vs. *M*_Incompatible_ = 5.829, *SD* = 1.774; *F*(1, 34) = 8.384, *p* = 0.007, $\eta_{p}^{2}$ = 0.198 [CI_95%_ 0.017–0.405]}. No differences in evaluations emerged when participants were not thirsty {*M*_Compatible_ = 4.774, *SD* = 2.012 vs. *M*_Incompatible_ = 5.194, *SD* = 2.197; *F*(1, 30) = 2.006, *p* = 0.167, $\eta_{p}^{2}$ = 0.063 [CI_95%_ 0.000–0.265]}.^4^

## General Discussion

The most important result in the present study is that the Stroop conflict task influenced individuals’ evaluations of products when those products were goal relevant.

Previous research showed a strong and robust main effect of conflict negativity on ambiguous and neutral stimuli (Dreisbach and Fischer, 2012; Fritz and Dreisbach, 2013; Goller et al., 2017; Damen et al., 2018). These studies were designed to assess the hedonic property of cognitive conflict, and as such provided important support for the notion of cognitive conflict as a negative signal. The fact that there was no main conflict negativity effect for the product stimuli in the present research suggests that the fundamental negativity emerging from cognitive conflict does not simply change our evaluations for actual products. A clear goal to interact with those products is required.

When participants were thirsty, both products were evaluated more favorably compared to when participants were not thirsty. This is in line with studies showing that goal-relevant stimuli are automatically evaluated more positively than irrelevant stimuli (e.g., Ferguson and Bargh, 2004). More importantly, when participants were thirsty, the Stroop conflict task changed the evaluations of the bottled water brands as a function of Stroop (non-)conflict. The product that had become associated with Stroop incompatibility – or conflict – was liked less; the product that had become associated with Stroop compatibility – or non-conflict – was liked more. The fact that this effect only emerged when the products were goal relevant is important information in the scarcely investigated topic on the consequences of cognitive conflict and conflict negativity: Goal or product relevance is likely required for our evaluations of meaningful items to change through cognitive conflict.

The moderating influence of goal and product relevance has been well documented in the social cognitive domain. Research on evaluative conditioning, for example, shows that EC is more effective when the associated items are relevant (Verwijmeren et al., 2012). Similarly, the literature on priming shows that primes are especially effective if the prime is relevant or applicable to the person’s current motivations (e.g., Higgins, 1996; Strahan et al., 2002). Of special interest is a study by Karremans et al. (2006) on subliminal soda brand priming. That study explored the degree to which the priming of Soda brands could influence product preferences and found that participants were only affected by the primed brand when they were thirsty – an outcome very similar to the one reported here. The present study adds to this literature by showing that Stroop conflict and non-conflict can influence product evaluations when those products are able to fulfill a goal.

Previous research from our laboratory showed compatible and incompatible Stroop items to strongly influence the evaluations of associated stimuli. For example, in a previous publication (Damen et al., 2018), 10 out of 11 studies showed the influence of conflict on evaluations. Such effects typically emerged quickly and required a relatively small number of pairings of Stroop (non-)conflict with the associated stimuli. The present study as such is a continuation in a line of studies using highly similar methods and may therefore be regarded as relatively reliable. However, this paper reports a single study, featured a relatively low number of trials, and the critical effect is only observed in one subgroup. Therefore, caution must be exercised when interpreting the results. Specifically, the exact importance of goal relevance requires more scientific exploration. For example, it is possible that given more trials (and therefore more pairings of conflict and non-conflict with stimuli), a main effect of cognitive conflict on liking may emerge independent from goal relevance – a logical path for future research to take.

Although the Stroop task is typically considered a task in which responses are required, a considerate number of studies have also explored the cognitive consequences of Stroop words merely being presented. Specifically, a line of studies (Dreisbach and Fischer, 2012; Fritz and Dreisbach, 2015) showed that incompatible Stroop primes led individuals to more quickly categorize negative targets as being negative – even without the requirement to respond. Similarly, compatible Stroop primes led individuals to more quickly categorize positive targets as being positive. Other research revealed the evaluations of neutral targets to be influenced by Stroop primes (Fritz and Dreisbach, 2013; Damen et al., 2018). Given that most real-life advertisements do not require interaction, it would be interesting to explore whether the evaluations of consumer products are similarly influenced through Stroop priming, either presented together with the products in repeated fashion, or even presenting a Stroop item on product packaging.

Given that the aim of this research was to explore the applicability of cognitive conflict on consumer evaluations, more studies are needed to establish the exact processes underlying the conflict negativity effects. For example, at the moment, it is unclear whether it is the conflict itself, or the upregulation of attention and/or conflict awareness that is driving conflict negativity. Some evidence for a direct effect of conflict comes from Fritz and Dreisbach (2015) who found that longer conflict prime durations led to weaker conflict negativity. However, no effect of prime duration was found in a study by Damen et al. (2018; Study 1A). Exactly, at which level conflict operates to influence evaluations is an important matter for future research to establish.

The origins of the Stroop effect have also been a matter of much scientific debate (MacLeod, 1991, 2005), ranging from perceptual incongruency, to response conflict (De Houwer, 2003), to contingency learning in the Stroop task’s typical setup (Crump et al., 2006; Schmidt, 2013; Braem et al., 2019). To address the influence of potential contingency learning, a future paradigm could employ two instead of four Stroop colors (Frings et al., 2018). Another possible avenue would be to explore the effects of other tasks of cognitive conflict that do not employ incompatible stimulus features (e.g., the Simon Task; Simon and Rudell, 1967). Finally, future research could explore potential congruency sequence effects (CSEs; Egner, 2007). As the intervals between Stroop targets in the present research were relatively large due to the presentation of product pictures, it was not appropriate to explore CSEs. A future study could however simultaneously present the Stroop targets with the product pictures thus allowing (the influence of) CSEs to be explored.

The Stroop effect is primarily considered to reflect interference on the incompatible trials. It is not – or to a much lesser extend – considered to emerge as a consequence of any response facilitation on the compatible trials (Stroop, 1935; MacLeod, 1991, 1998). In similar vein, conflict negativity is typically considered to drive changes in affect (e.g., Botvinick, 2007; Dreisbach and Fischer, 2015). A recent publication however showed that stimuli were liked more when they had become associated with the compatible non-conflict trials on a Stroop task (Study 4; Damen et al., 2018). Such a result would be in line with the literature on processing and perceptual fluency (Reber et al., 1998, 2004), suggesting that when stimuli are more easily perceived and more easily processed, they are typically also liked more. The exact nature and limits of a potential Stroop positivity effect remain to be carefully explored (Damen et al., 2018, p. 19; also see Schouppe et al., 2015; Ivanchei et al., 2021); however, whether it could be applied to positively change evaluations is an interesting avenue for future research.

## Conclusion

Conflict experiences are omnipresent and pervasive in everyday human life. In the present research, we show that when goal-relevant, the conflict and non-conflict events emerging from the Stroop task are capable of changing individuals’ evaluations of actual products.

## Data Availability Statement

The raw data supporting the conclusions of this article will be made available by the author, without undue reservation.

## Ethics Statement

The studies involving human participants were reviewed and approved by the Faculty Ethics Assessment Committee – Social Sciences, Utrecht University. The patients/participants provided their written informed consent to participate in this study.

## Author Contributions

The author confirms being the sole contributor of this work and has approved it for publication.

### Conflict of Interest

The author declares that the research was conducted in the absence of any commercial or financial relationships that could be construed as a potential conflict of interest.

## Footnotes

^1^In fact, in Europe, it was only available in the United Kingdom and only between February and March 2004.

^2^Whether or not participants erred did not bear relevance to the statistical conclusions.

^3^Measuring thirst at the end of study has methodological limitations (e.g., thirst may be influenced by the task itself). However, asking individuals to rate their thirst earlier is likely to make thirstiness highly accessible (leading to goal priming). And asking participants about thirst earlier may also lead participants to believe that the study was on which product was more thirst quenching. The aim was to avoid such deliberative considerations.

^4^In a variety of analyses, the relation between performance on the Stroop task and subsequent evaluations was explored. No such relations were found (all *p*’s > 0.90).
